# Stiffness after unicompartmental knee arthroplasty: Risk factors and arthroscopic treatment

**DOI:** 10.1051/sicotj/2021034

**Published:** 2021-05-19

**Authors:** Gaspard Fournier, Romain Gaillard, John Swan, Cécile Batailler, Sébastien Lustig, Elvire Servien

**Affiliations:** 1 Department of Orthopedic Surgery and Sport Medicine, Croix-Rousse Hospital, FIFA Medical Center of Excellence 69004 Lyon France; 2 Univ Lyon, Université Claude Bernard Lyon 1, IFSTTAR, LBMC UMR_T9406 69622 Lyon France; 3 EA 7424 – Interuniversity Laboratory of Human Movement Science, Université Lyon 1 Lyon France

**Keywords:** Unicompartmental Knee Arthroplasty, UKA, Stiffness, Arthroscopic treatment, Arthrolysis

## Abstract

*Introduction*: One of the principal complications after total knee arthroplasty (TKA) is stiffness. There are no publications concerning stiffness after unicompartmental knee arthroplasty (UKA). Study objectives were to describe the incidence of stiffness after UKA, to look for risk factors, and to describe safe and effective arthroscopic treatment. *Methods*: There were 240 UKA performed between March 2016 and January 2019 included. Robotic-assisted surgery was performed in 164 patients and mechanical instrumentation in 76 patients. Stiffness was defined as flexion < 90° or a flexion contracture > 10° during the first 45 post-operative days. Patients with stiffness were treated with arthroscopic arthrolysis. Several factors were studied to look for risk factors of stiffness: body mass index, gender, age, mechanical or robotic instrumentation, preoperative flexion, previous meniscectomy, and anticoagulant treatment. Arthrolysis effectiveness was evaluated by flexion improvement and UKA revision rate. *Results*: 22 patients (9%) developed stiffness. Mechanical instrumentation significantly increased the risk of stiffness with OR = 0.26 and *p* = 0.005. Robotic-assisted surgery decreased the risk of stiffness by five-fold. Before arthrolysis, mean knee flexion was 79°, versus 121° (53% improvement) after arthroscopic arthrolysis. Only 2 patients (9%) underwent UKA revision after arthrolysis. *Discussion*: Stiffness after UKA is an important complication with an incidence of 9% in this study. Arthroscopic arthrolysis is a safe and effective treatment with a range of motion improvement of > 50%. Robotic-assisted surgery significantly decreases the risk of postoperative stiffness.

*Level of evidence*: Level III, therapeutic study, retrospective cohort study

## Introduction

Unicompartmental knee arthroplasty (UKA) is a common treatment for partial knee osteoarthritis. Outcomes of UKA have improved in recent years, likely due to improved surgical technique and superior implant designs. Performing UKA with robotic assistance results in better implant positioning and decreased complications [1, 2]. Numerous publications are showing that UKA has a decreased complication rate and superior postoperative results compared to total knee arthroplasty (TKA) [3–8]. Complications of UKA include osteoarthritis progression, aseptic loosening, bearing dislocation, and pain [9, 10]. For these complications, the recommended treatment is a revision to TKA, which is a difficult surgery. This may explain why some surgeons prefer to primarily perform TKA.

Stiffness is a common complication of TKA, with a reported incidence of between 1% and 13% postoperatively [11, 12]; treatments performed include manipulation under anesthesia, arthrolysis arthroscopically or open, and revision TKA. Stiffness after TKA is defined as flexion < 90° or a flexion contracture > 10° (lack of extension). We define early stiffness at < 45 days post-TKA, late stiffness < 6 months postoperatively, and chronic stiffness that lasts for > 6 months.

Stiffness is not frequently reported in UKA. Only a few studies report this complication [13, 14], and there is no literature on stiffness and risk factors after UKA. The objectives of this study were to report the incidence of stiffness after UKA, in the first three postoperative months, then, to investigate risk factors for stiffness, and finally to describe and analyze arthroscopic treatment and its effectiveness.

Hypothesis was that robotic instrumentation decrease the rate of stiffness versus mechanical instrumentation and that arthroscopic debridement is a safe and efficient treatment of stiffness after UKA.

## Materials and methods

### Population

This single-centre retrospective study was based on prospectively collected data of a cohort of 240 consecutive primary UKAs performed at our department between March 2016 and January 2019 by two senior surgeons. Inclusion criteria were all patients who underwent a UKA in that study period with a minimum follow-up of 1 year.

**Figure 1:**
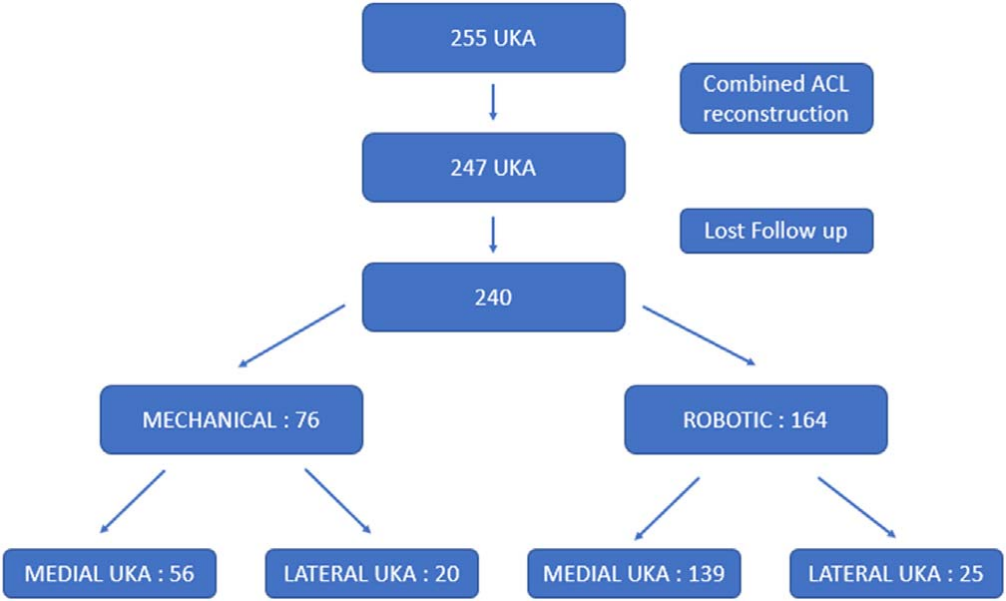
Flowchart.

Exclusion criteria were patients with previous knee fracture, previous osteotomy, anterior cruciate ligament reconstruction, or sagittal instability before surgery to decrease the risk of UKA failure (Figure 1). Characteristics of the final population of 240 patients are summarized in Table 1.

**Table 1:** Final study population characteristics *SD standard deviation, Min minimum, Max maximum*.

	Population
Total Population	240
Male	94
Female	146
Age (mean ± sd) [Min; Max]	65 ± 9 [27;93]
BMI (Kg/m^2^) (mean ± sd) [Min; Max]	27 ± 4 [17;36]
Antiplatelet agents	26 (10%)
Anticoagulant	11 (4%)
Robotic surgery	164 (68%)
Medial UKA	139
Lateral UKA	25
Mechanical surgery	76 (32%)
Medial UKA	56
Lateral UKA	20
Lateral meniscectomy	20 (8%)
Medial meniscectomy	64 (26%)
Preoperative flexion (°) (mean ± sd) [Min; Max]	128 ± 8 [90; 150]

### Ethical considerations

All procedures were performed by the ethical standards of the institutional and/or national research committee, the 1964 Helsinki declaration, and its later amendments, or comparable ethical standards.

### Operative technique

**Figure 2:**
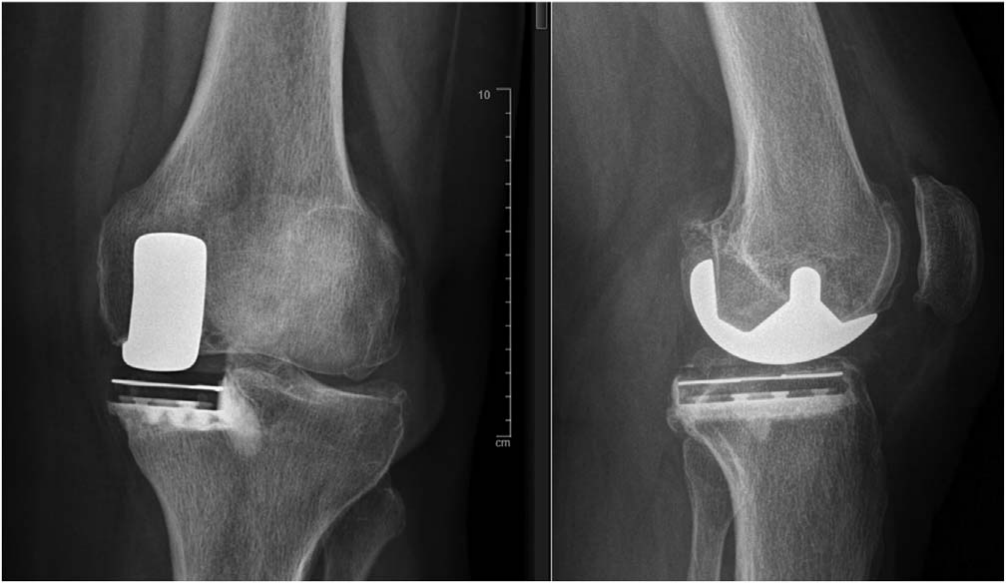
Medial HLS Uni Evolution™ UKA (AP view and profile).

The medial mid-vastus approach was systematically used for medial UKA and the lateral parapatellar approach was used for lateral UKA. Two different implants were used, and two different instrumentations were used: mechanical instrumentation or robot-assisted surgery. The first type of implant used was a cemented resurfacing unicompartmental prosthesis with an all-polyethylene tibial component (HLS Uni Evolution™, Corin^®^) (Figure 2).

**Figure 3:**
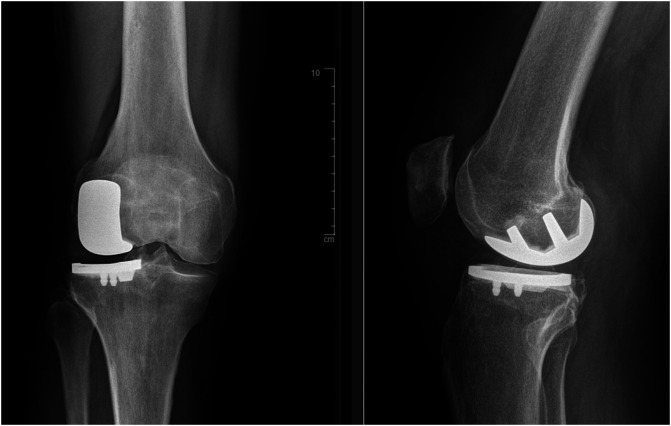
Medial Journey™ UKA, (AP view and profile).

The second type of implant used was a cemented resurfacing unicompartmental prosthesis with a metal-backed tibial component (Journey™ Uni unicompartmental knee system, Smith and Nephew^®^) (Figure 3).

The BlueBelt Navio robotic surgical system (Smith and Nephew^®^) was used in robotic-assisted surgery [15]. The surgical robotic-assisted or conventional techniques have already been described [16]. The distribution of the two different implants among the study population is shown in Table 2.

**Table 2:** Distribution of implants and surgical technique.

	Mechanical instrumentation	Robotic assisted surgery	Total
HLS Uni Evolution, Corin^®^	43	43	86
Journey™ Uni unicompartmental knee system, Smith and Nephew^®^	33	121	154

### Follow-up and evaluation

Patients underwent clinical and radiological evaluation at 21 days, 45 days, 3 months, 1 year postoperatively, and then every year thereafter. The initial diagnosis of stiffness was made clinically during the first 45 days postoperatively. Radiographic examination was performed at each consultation (including weight-bearing anteroposterior and lateral knee radiographs, patellar axial view, and full-length standing radiographs).

### Arthroscopic treatment of stiffness

The treatment of stiffness consisted of arthroscopic arthrolysis. Two standard anterior portals were made: inferolateral and inferomedial, and only if necessary, a third superolateral portal was made. A step-by-step approach was used as described by Volchenko et al. [17] for TKA arthroscopic arthrolysis. The first step was debridement of the patellofemoral and suprapatellar compartments. Then, medial and lateral gutter synovectomy and lysis of adhesions were performed. Then, medial and lateral capsular release was practiced. To finish, an anterior interval release with intercondylar notch examination and debridement was performed. Bacteriological samples were systematically performed to exclude sepsis as a cause of stiffness.

### Postoperative management

Patients underwent physiotherapy with no restrictions of flexion. Continuous passive motion with Kinetec Spectra Essential™ (Kinetec^®^) was performed for 20 h per each 24 h during immediate 7 days after surgery. No prophylactic thromboembolism anticoagulants were administered to reduce the risk of hematoma and adhesions reforming. Patients underwent clinical and radiological evaluation at 21 days, 45 days, 3 months, and 1 year postoperatively and then every year thereafter.

### Evaluation of treatment effectiveness

Arthroscopic arthrolysis was considered effective if flexion was > 90° after 6 months and if there was no revision surgery.

### Statistical analysis

Continuous variables were averaged and reported with standard deviation and extremes. The multinomial logistic regression model was used to investigate the relationship between stiffness and patient risk factors.

A *P*-value < 0.05 was considered statistically significant in each analysis. Statistical analyses were performed using XLstat (2015.1 version, Addinsoft, France).

## Results

### Stiffness

Firstly, stiffness incidence has been evaluated: 22 patients (9%) developed stiffness (Table 3).

**Table 3:** Stiffness incidence and risk factors/*ns: no significant, OR: odds ratio*.

	Stiffness	No Stiffness	OR (*p*)
Patient	**22 (9%)**	218 (91%)	
Age	61	66	0.98 (ns)
BMI (Kg/m^2^)	26	27	0.93 (ns)
Preoperative flexion (°)	127.5	129.1	0.98 (ns)
Meniscectomy antecedent	14	72	2.4 (*p* = 0.07)
Mechanical instrumentation	14	62	**0.26 (*p* = 0.005)**
Antiplatelet agents	0	27	0.17 (ns)
Anticoagulant	1	10	2.3 (ns)
Postoperative flexion (°)	79	126.9	

### Risk factors (Table 3)

At a second time, risk factors to present stiffness have been evaluated.

Different risk factors studied were body mass index (BMI), age, mechanical versus robotic instrumentation, meniscectomy antecedent, preoperative flexion, taking anticoagulant, or not and taking antiplatelet agents or not and kind of implant. Results are summarized in Table 3.

Only one risk factor studied seemed to have a significant impact on stiffness: mechanical instrumentation (*p* = 0.005) with odds ratio, OR = 0.2. Thus, using robotic instrumentation reduces the risk of stiffness by > 5 times after UKA. Another interesting risk factor with an important OR was found: meniscectomy antecedent (OR = 2.4). The difference was no significant, but patients with meniscectomy antecedent have twice the risk to present stiffness.

### Arthrolysis effectiveness

Finally, the effectiveness of arthroscopic treatment was evaluated. Before arthrolysis, the average knee flexion was 79° comparing to 121° after arthrolysis. The average increase in range of motion was 40° (53% improvement). Only 2 patients (9%) had a failure of arthrolysis and they were revised to a TKA.

## Discussion

The most important finding of this study was that stiffness is an important complication after UKA, with 9% of the patients in this cohort presenting with stiffness after UKA. Arthroscopic debridement is an effective treatment with only 9% of patients still requiring revision to TKA after arthrolysis. Performing robotic-assisted UKA surgery significantly reduced the risk of stiffness by > 5 times (*p* = 0.005). Patients with meniscectomy antecedent present twice the risk to have stiffness after UKA.

After TKA, stiffness is a common complication. Multiple treatments are recommended: manipulation under anesthesia, arthroscopic arthrolysis, open surgery. Volchenko et al. [17] described a benefit to practicing arthroscopic lysis of adhesions associated under anesthesia (with 50% improvement) compared with manipulation under anesthesia alone on a cohort of 70 patients with stiffness after TKA. This improvement is similar to our study findings, in which there was a 53% improvement in knee flexion.

Regarding UKA, manipulation under anesthesia can be effective but may result in complications such as fracture, rupture of the extensor mechanism, or chondral damage [18]. Manipulation under anesthesia is only performed for stiffness after TKA, to avoid the risk of chondral damage in patients with UKA. However, performing a second invasive procedure may increase the risk of complications for patients, such as anesthesia complications, surgical complications, and periprosthetic sepsis compared to simple manipulation under anesthesia.

Hurst et al. [14] described arthroscopic evaluation after UKA for several complications. They found a stiffness rate of 12% after UKA, which is similar to our study. They concluded that arthroscopic evaluation after UKA is a safe method without any complication and effects on the implant survival rate. In our study, no patients had complications due to arthroscopy.

In our study, the robot seems to reduce the risk of stiffness. Several causes can explain this result.

Firstly, robotic-assisted surgery seems to improve implant positioning. Batailler et al. [2] had already shown that using robotic-assisted surgery increased the accuracy of implant positioning and decreased the revision rate. It is likely for those reasons that patients with robotic-assisted surgery have a lower rate of stiffness.

Indeed, Herry et al. [16] have shown better joint line restitution using robotic-assisted surgery during UKA. Simpson et al. [19] demonstrated that after UKA, mean strain on the proximal tibial cortex increased by 6, 13, and 18%, respectively, when tibial resection levels of 2, 4, and 6 mm were modeled, showing the importance to restore joint line-height to decrease pain and potentially stiffness after UKA. Robotic-assisted surgery appears to be superior by improving implant positioning and reducing complications after UKA.

The use of the robot allows better restitution of the physiological kinematics of the knee and this may explain the best post-operative follow-up and therefore the reduction of the risk of stiffness.

Then, several studies have shown a decrease in postoperative pain with the use of the robot [20, 21]. This may also explain the decrease in the rate of stiffness in patients operated with robotic.

Postoperative management is a very important point for us after stiffness treatment.

After primary TKA, continuous passive motion does not provide an additional benefit compared to traditional physiotherapy [22]. However, after surgery for stiffness, we believe that it assists patients in recovering self-confidence by allowing the patient to visualize the flexion improvement and help them to maintain the improvement over the first seven days. Leijtens et al. [23] have shown a high rate of bleeding complication after TKA and UKA for patients who are administered anticoagulant treatment. For this reason, no patients received anticoagulant treatment after arthroscopic debridement to decrease the risk of hematoma and stiffness. No patients had thrombo-embolism in our study.

It is important to note that there is very little literature reporting upon stiffness after UKA. There are numerous studies concerning stiffness after TKA, but not for UKA. This is surprising given that the rate of stiffness is around 9%, which is not much lower than a 12% stiffness rate after TKA. Stiffness after UKA is an important complication that deserves closer evaluation.

There are some limitations to this study that need to be acknowledged. By nature of the retrospective study design, some degree of bias could have been introduced, since patients were not randomized and indication bias for the use of robotic assistance surgery versus mechanical instrumentation may have occurred. Then, the short follow-up after arthroscopic treatment may overestimate the effectiveness of arthroscopic arthrolysis, however, the literature tells us that most stiffness occurs within the first two months.

## Conclusions

Stiffness after UKA is an important complication with an incidence of 9% in this study. Arthroscopic arthrolysis is a safe and effective treatment with a range of motion improvement of > 50%. Robotic-assisted surgery significantly decreases the risk of postoperative stiffness.

## Conflict of interest

Prof. Sébastien Lustig is a consultant for Medacta, Heraeus, Corin, Amplitude, Groupe Lépine, Depuy, Smith & Nephew, Stryker. Prof. Sébastien Lustig receives support from Corin and Amplitude. Prof. Sébastien Lustig is a board member of KSSTA, Maitrise Orthopédique. The other authors declare that they have no conflicts of interest.

Prof. Elvire Servien is a consultant for Corin and Smith & Nephew.
